# Multidimensional Family Therapy for Justice-Involved Young Adults with Substance Use Disorders

**DOI:** 10.1007/s11414-023-09852-5

**Published:** 2023-08-02

**Authors:** Howard A. Liddle, Gayle Dakof, Cynthia Rowe, Adhar Bashatu Mohamed, Craig Henderson, Trenten Foulkrod, Megan Lucas, Michael DiFrancesco

**Affiliations:** 1 University of Miami Miller School of Medicine, Miami, USA; 2 Sam Houston State University, Huntsville, USA; 3 MDFT International, Inc., Miami, USA

**Keywords:** Substance use, Criminal justice, Young adults, Emerging adults/transitional age youth (TAY), Multidimensional family therapy (MDFT)

## Abstract

The present study explored the acceptability, feasibility, fidelity, and outcomes of a young adult adaptation of multidimensional family therapy (MDFT), an evidence-based family treatment originally developed for adolescents. Participants included 22 individuals between the ages of 19 to 25 who were enrolled in a criminal drug court program. MDFT was found to be feasible and was delivered with strong fidelity to young adults and their families. Participants reported high satisfaction with MDFT, and 95% completed treatment. Analyses revealed statistically significant decreases in substance use on all indicators from baseline to the 6-month follow-up. Significant improvements were also noted in vocational functioning, including a 73% increase in full-time employment from baseline to 6-month follow-up. Criminal justice outcomes included a significant decrease in legal risk, and 86% of study participants had no rearrests from baseline through the 18-month follow-up period. The article concludes with recommendations for implementing family-based interventions with young adults, as well as future research directions in this important area.

## Introduction

Young adults between the ages of 18 and 25, also referred to as emerging adults or transitional age youth, traverse a significant developmental period that is distinct from both adolescence and later adulthood.^1–5^ Making the successful transition into adult work and family roles is essential for healthy and fulfilling development, and tends to be influenced by both positive and negative factors set in motion during childhood and adolescence. Young adulthood is thus a period of both risk and opportunity that can overwhelm those with early life challenges such as substance misuse, unstable mental health, juvenile justice involvement, and troubled family relationships. These young people are at a serious developmental disadvantage as they transition into adulthood.^6^

An expanding body of research indicates that young adults have higher rates of mental health and substance use disorders, arrests, and recidivism than either adolescents or adults ages 26 and older.^7–11^ They are also less likely to access needed services due to gaps between the pediatric and adult treatment systems, inconsistent insurance coverage, and other challenges.^8^ For some young adults, substance use and mental health disorders may only be addressed when contact is made with the criminal justice system, which arguably is not equipped to provide the highest quality treatment.^10,12^ As a result, the treatment of young adults is often characterized by high dropout rates and poor clinical outcomes.^13–15^ Moreover, interventions are rarely designed to address the distinctive developmental needs of young adults with substance use and mental health disorders.^16,17^ Young adults are generally treated with older adults with few adaptations or considerations for their unique stage of life.^16^ Simultaneously, there is growing recognition that the family is an important target of successful treatment for mental health and substance use disorders, not only with adolescents but also with adults.^10,13,18–22^ Decades of research confirm the reciprocal relationship between family functioning and substance use. Substance use and mental health disorders in young adults present challenges to family members, especially to parents.^18,23–25^ Parents and concerned significant others frequently provide financial support and housing to the young adults in their lives and facilitate their access to behavioral health care.^8,13^ The burden of caring for family members with substance use and mental health disorders increases risk for health problems among caregivers.^21^

While treatment for young adults may engage with family members, there is an important difference between “family-involved treatment” and “family therapy.” Family involvement alone may not be sufficient to transform the lives of young adults with serious substance use and mental health challenges. Family-involved treatments mainly provide psychoeducation and encourage family members to support the young adult’s recovery.^18^ In treating young adults, “family involvement” may include romantic partners and friends without involving parents in the change process.^10,17^ Family therapy, on the other hand, seeks to change the whole system, including parents and others who are influential in the young person’s life. These approaches recognize that while developing close relationships with romantic partners and peers is essential to healthy development, parents maintain powerful influence into young adulthood and beyond.^26^ In fact, young adults are now in closer contact with their families than those from previous generations.^8^ For example, approximately 52% of 18- to 29-year-olds live with their parents.^27^ For these reasons, parents of young adults may play an important role in promoting change as an integral focus in family therapy with young adults.

Despite the effectiveness of family therapy for adolescent problems, no recent research has investigated its potential with young adults and their parents. Over 40 years ago, Stanton and Todd^26^ published pioneering work on family treatment for adult substance use disorders. Structural-strategic family therapy was shown to be effective with 46 young adults (average age of 25 years) and their parents. These results set the foundation for programs of research on couples therapy for adult substance misuse and family treatments for adolescent substance use.^18,28^

In several published randomized clinical trials focusing on adolescents, multidimensional family therapy (MDFT) has been shown to reduce substance use, delinquency, and mental health problems, and to improve school performance and family functioning.^29–36^ MDFT’s approach is collaborative, comprehensive, and family-centered. It simultaneously addresses substance use, behavior problems and criminality, mental health, violence and aggression, educational challenges, and family functioning. MDFT works in four areas: the client (adolescent or young adult), the parents, the family (client and parent/family together), and the community. Thus, one therapist, in an integrated fashion, provides individual youth sessions, parent sessions, family therapy, and also links family members to community services. It is flexible and adaptable, with strong effects for adolescents in justice settings.^37^ Independently conducted economic analyses support the feasibility of MDFT implementation.^38,39^ Moreover, from its inception, MDFT has been guided by developmental psychology and developmental psychopathology,^40–42^ making it a promising model for adaptation to various developmental periods. The present study builds on the robust and consistent evidence supporting MDFT in the treatment of adolescentss^43^ to evaluate the potential of this family approach with young adults.

### Current Study

The current study is built upon four foundations that indicate that families generally—and parents in particular—remain untapped as potential resources in the treatment of young adults. First, families are integral to young adult functioning. Second, many young adults today have regular contact with or live with their parents. Third, pioneering research validated family treatment with young adults and their parents over 40 years ago. Fourth, family-based treatments are among the most effective for adolescent substance use and behavior problems.

In the present study, the authors hypothesized that MDFT for young adults would be (a) feasible and acceptable to young adult participants and their parents, as evidenced by high treatment enrollment, participation and completion, and high treatment satisfaction; and (b) participants would reduce substance use and criminality and show improvement in vocational or educational functioning from baseline through the follow-up period.

## Methods

### Participants

This study was implemented in the State of Florida 11^th^ Judicial Circuit Criminal Court in Miami Dade County. All young adults ages 18 to 25 who were adjudicated to the Miami Dade Criminal Drug Court and had family living in South Florida were eligible to participate. Miami Dade Criminal Drug Court eligibility were as follows: (a) at least one open non-violent third-degree felony charge, excluding sale or delivery of a controlled substance (or with intent to sell/deliver a substance); (b) no more than two prior felony convictions or withholds of adjudication; (c) no prior conviction or withhold of adjudication for any forcible felony; and (d) a substance use disorder diagnosis.

### Procedures

The University of Miami Institutional Review Board (IRB) approved and monitored the study. Twenty-five consecutive admissions into criminal drug court meeting study eligibility criteria were identified by drug court staff who briefly described the study and asked the young adults if they could give their contact information to University of Miami research staff. Research staff then contacted potential participants to describe the study in detail and solicit informed consent. Twenty-two provided their informed consent to participate, yielding an 88% response rate.

Self-report data from young adult participants were collected at baseline and again at 6 months following baseline by University of Miami research staff. The drug court judge and drug court staff were blind to study participation. Once the intervention phase of the study was completed, drug court staff extracted arrest data from baseline to 18 months post-baseline from a criminal justice database maintained by the State of Florida. Treatment data were gathered from treatment provider records.

### Setting and context

Study participants were adjudicated in a single drug court with one judge presiding. Young adults were expected to follow criminal drug court requirements, namely successfully participating in substance use treatment, attending court hearings, and complying with court orders. The court incorporated the key components of drug court and best practice standards as defined by the National Association of Drug Court Professionals.^44,45^ These standards were originally created in 1996 and have been validated over subsequent years. The 10 key components and standards are composed of the following: (1) target population; (2) equity and inclusion; (3) roles and responsivities of the judge; (4) incentives, sanctions, and therapeutic adjustments; (5) substance use disorder treatment; (6) complementary treatment and social services; (7) drug and alcohol testing; (8) multidisciplinary team; (9) census and caseloads; and (10) monitoring and evaluation.^44,45^ Research findings indicate that drug court outcomes are related to adherence to these principles and standards.^46,47^

As is standard practice in drug courts (standard # 5), all drug court participants were required to participate in substance use treatment. In the Miami Dade Criminal Drug Court, participants were referred to various community-based substance use treatment providers offering a variety of treatment approaches (e.g., 12 step-based group treatments, cognitive behavioral therapy, and MDFT), and levels of care (i.e., outpatient, intensive outpatient, and residential treatment). A single community-based agency, Concept Health Systems (CHS), provided MDFT to all study participants. Once referred to MDFT, participants did not receive additional drug treatment services.

### Treatment

MDFT is a comprehensive, developmentally oriented family-based treatment.^37,48^ Therapists work individually in four intervention domains: young adult, parent, family, and community. According to treatment stage and goals, clinicians have sessions alone with the young adult, alone with the parents, and with the young adult and parents together. This format supports combinations of intrapersonal, interpersonal, and family transaction (family relationships) therapy change mechanisms and methods. Treatment averages 4 to 6 months. A single clinician works with the young person and their family members in both clinic-based and in-home sessions. In the community domain, therapists attend meetings at court and advocate for the young adults and family members as needed for school, job placement, and other case management needs.

In the young adult adaptation of MDFT, therapeutic principles, orientation, structure, and components of MDFT are the same as the adolescent version, apart from certain changes in the format and content of sessions. In the young adult version, sessions may be conducted with the young adult and their intimate partners and others in addition to their parents, siblings, and extended family. In comparison to the adolescent version of MDFT, there are more individual sessions alone with the young adult. The content of all sessions, as befits the developmental level of the young adult and family, is decidedly different from certain content in adolescent MDFT. For example, in MDFT for young adults, issues of job and career, intimate relationships, and sexuality take on increased significance in their daily lives. Some young adults are also parents themselves, and thus, the MDFT therapist helps them succeed in their parenting role. Parents of young adults frequently need help to transition from parenting a teenager to a young adult, including how to hold expectations in a context of mutual respect. As in MDFT with adolescents, family sessions focus on enhancing communication, understanding, family problem solving, deepening the emotional connection among family members, and helping young adults and parents renegotiate their relationships. MDFT protocols detail the key interventions, and a modular treatment design helps to organize multiple interdependent therapy targets and prescribed therapeutic techniques.

### Treatment fidelity

Adherence to MDFT techniques was assessed using the Multidimensional Family Therapy Intervention Inventory (MII),^49^ which measures the essential interventions of MDFT. The MII has been used extensively in MDFT clinical supervision, training, and randomized clinical trials, and has demonstrated strong interrater reliability.^50^ Independent raters view video recordings and evaluate therapy sessions on the extensiveness of 16 core MDFT interventions using a seven-point Likert-type rating scale ranging from 1 (not at all) to 7 (extensively). Based on more than 650 previous MII ratings with adolescent clients and their families, a score of 3.0 or higher represents the benchmark of adequate adherence.^50^ One session from 6 of the 22 cases (27%) was randomly selected and rated on the MII. Ratings of session videos showed that MDFT therapists achieved an average adherence score of 3.3 across sessions (SD = 0.8), which met the established benchmark of adequate adherence.

To evaluate therapeutic fidelity to treatment parameters, therapeutic contact data (session composition and length) were gathered from the clinical provider. Study participants received an average of 9.25 h of therapy per month (a little over 2 h of therapy per week) over the course of 5.96 average months of treatment. Fifty-one percent of sessions were held alone with the young adult. Twenty-eight percent of sessions were family sessions, 16% were sessions with the parents without the young adults, and 5% of the sessions were in the community domain (e.g., visits to colleges or vocational training programs). These data are consistent with MDFT benchmarks.

### Measures

#### Substance use

Two measures were used to assess substance use: The Addiction Severity Index (ASI)^51^ and the Timeline Follow-Back Method (TLFB).^52^ The ASI is a semi-structured interview that assesses the participant’s past and current functioning. It is a widely used instrument that has shown excellent reliability and validity.^53,54^ In this study, the Alcohol Use and Drug Use Composite Scores were analyzed. The TLFB method measures substance use frequency and consumption. The measure has been widely used in substance use treatment studies with adults and adolescents.^55–57^ The TLFB obtained 90-day retrospective reports of daily substance use. In this study, we analyzed number of days using any drug and number of days participants reported drinking alcohol to intoxication.

#### Criminal justice/legal status

Arrest data were extracted from the criminal justice database maintained by the State of Florida for each participant from baseline through 18 months post-baseline. The number of arrests and type of charge (felony or misdemeanor) were calculated for 3 time periods: (1) baseline to 6 months after baseline, (2) 6 months to 12 months after baseline, and (3) 12 months to 18 months after baseline. The ASI Legal Composite score, which measures legal involvement (e.g., presently awaiting charges, number of days of illegal activity involvement, self-assessment of seriousness of legal problems), was also used in analyses.

#### Vocational functioning

Researchers used three measures of vocational functioning. Developmental competence was assessed using the Work subscale from The Status Questionnaire.^58,59^ The Work subscale assesses attitudes about work, completion of job responsibilities including getting to work on time, doing one’s job, and absences. The employment scale from the ASI was also used, which includes items such as possession of a valid drivers’ license and access to a car, days worked for pay, and amount earned. Employment status (unemployed, employed part time, employed full time) was gathered from provider records coded as follows: (0) = employed full time, (1) = employed part time, (2) = actively seeking work but not currently employed, and (3) = not employed and not actively working towards employment.

#### Client satisfaction

Participant satisfaction with MDFT was measured by the Service Satisfaction Scale (SSS-16),^60^ an instrument designed to measure several components of satisfaction with behavioral health services (provider manner and skills, perceived outcome, procedures, and accessibility). This study used the total satisfaction score derived from all items. Young adults completed the SSS-16 at 6 months.

#### Data analytic approach

Given the small number of participants, non-parametric *t*-tests for related samples, specifically the Wilcoxon signed-rank test, were used to compare outcomes between baseline and the two follow-up periods. Non-parametric tests are used as an alternative to parametric tests (e.g., repeated measures analysis of variance) that rely on a hypothetical sampling distribution on which probability values are derived and a determination is made regarding the statistical significance of the test. A non-parametric test is considered “distribution free,” removing many of the assumptions of traditional parametric testing. It is often the most appropriate technique for repeated measures with small samples. The Wilcoxon signed-rank test focuses on the order or ranking of scores rather than making assumptions pertaining to a continuous distribution underlying the data.

Along with results of the non-parametric statistical tests, effect sizes are reported, which are appropriate in small sample research, as they are unconfounded with sample size. The standardized mean difference (Cohen’s *d*) as a measure of effect size was used. Researchers first examined changes between baseline and 6-month follow-up, and then investigated the longer-term follow-up period (7 to 18 months post-baseline).

Analyses were conducted using SPSS Version 25.

## Results

### Sample characteristics

Twenty-five participants were approached for study enrollment. Three refused to participate, yielding an 88% response rate. Twenty-two young adults participated in the study. As presented in Table 1, 73% were between the ages of 18–20, and 27% were between 21 and 25. Seventy-one percent identified as male, and 29% as female. Participants self-reported their ethnicity as Hispanic (71%), African American (27%), and other (5%). All young adults had at least one open felony charge and a substance use disorder diagnosis. Notably, all the young adults who enrolled in the study had at least one of their parents or caregivers agree to participate in MDFT.

**Table 1:** Characteristics of participants in the sample

	*n*	%
Gender		
Male	15	71
Female	7	29
Age		
18–20	16	73
21–25	6	27
Race/ethnicity		
Hispanic	15	71
African American	6	27
Other	1	5
Substance use diagnosis	22	100
Felony charge	22	100

#### Attrition rates

The data capture rate was 95.5%, with 6-month follow-up data missing from only one participant. This participant was unavailable for the in-person post-treatment assessment. Two outcome indicators—rearrests and drug court graduation—were obtained for all study participants.

### Treatment satisfaction

Young adults were asked to report on their satisfaction or lack thereof with MDFT on the SSS-16. All participants indicated that they were “delighted,” the highest level of satisfaction on the SSS-16, with the treatment overall. Additionally, 100% of participants reported that they were “delighted” with most of the items related to the treatment, including “professional knowledge and competence of the therapist” and “ability of your therapist to listen to and understand your problems.” The lowest satisfaction scores were reported for “publicity or information about programs and services offered,” with 82% indicating that they were “delighted” and the remainder (18%) being “mostly satisfied.”

### Baseline to 6 months following baseline

Results are reflected in Table 2. Both self-report and criminal justice record data were available for this time period. As hypothesized, young adults who received MDFT showed significant reductions in substance use and offending, and significant improvement in vocational functioning from baseline to 6-month follow-up. With respect to substance use, the ASI Drug Use Composite (*Z* = − 4.02 *p* < .001, *d* = 1.87), Alcohol Use Composite (*Z* = − 3.36, *p* = .001, *d* = .83), TLFB Days Used Any Drug (*Z* = − 3.98, *p* < .001, *d* = 3.84), and Days of Alcohol Intoxication (*Z* = − 3.47, *p* ≤ .001, *d* = .71) all showed statistically significant decreases from baseline to 6-month follow-up with corresponding medium to large effect sizes.

**Table 2:** Baseline and 6-month central tendency measures, Wilcoxon tests, and effect size for self-report data

Variable	Mean BL (SD)	Mean 6 Mo (SD)	Median BL	Median 6 Mo	Wilcoxon test	*p*	Effect size^a,b^
TLFB Days Used Any Drugs	79.77 (19.38)	5.19 (19.52)	90	0	− 3.98	< .001	3.84
TLFB Days Alcohol Intoxication	15.50 (21.56)	0.29 (0.64)	6.50	0	− 3.47	.001	.71
ASI Alcohol Use Composite	0.11 (0.12)	0.01 (0.03)	0.09	< 0.01	− 3.36	.001	0.83
ASI Drug Use Composite	0.17 (0.08)	0.02 (0.04)	0.18	< 0.01	− 4.02	< .001	1.87
ASI Legal Composite	0.52 (0.14)	0.40 (0.18)	0.50	0.37	− 3.07	.002	0.86
ASI Employment Composite	0.07 (0.36)	− 0.01 (0.42)	0.05	− 0.17	0.22	.826	0.22
DCS Work	9.04 (1.56)	10.90 (1.79)	9.00	11.00	157.00	.002	1.19

Effect size is the standardized mean difference and is based on sample means rather than medians

^a^*TLFB* timeline follow-back, *ASI* Addiction Severity Index, *DCS* Developmental Competence Scales; *TLFB*, number of days used over 90-day period

^b^Small = .30, medium = .50, large = .70

Examination of criminal justice records indicated that only one participant was arrested during this period. Consistent with criminal record data, the ASI Composite Legal Scale showed a statistically significant decrease and a large effect size in legal risk from baseline to 6-month follow-up (*Z* = − 3.07, *p* = .002, *d* = .86).

Finally, with respect to vocational functioning, the Work subscale of the Status Questionnaire (SQ) Developmental Competence Scale showed a statistically significant increase in positive vocational functioning from baseline to 6 months after baseline (*Z* = 3.14, *p* = .002, Cohen’s *d* = 1.19). The ASI Composite Employment subscale did not show significant improvement from baseline to 6-month follow-up (*Z* = 0.22, *p* = .826, Cohen’s *d* = .22). However, the data indicated significant improvement in employment status from baseline to 6 months after baseline (*Z* = − 2.28, *p* = .023); 45% were unemployed at baseline versus 18% unemployed 6 months later. Additionally, 68% of the young adults were employed full time 6 months after baseline, which is a 73% increase in full-time employment from intake to 6 months later.

### Seven to eighteen months after baseline

Only criminal justice record data were available for this time frame. Twenty participants (91%) graduated from drug court. Between 6 and 12 months after baseline, two young adults (9%) were arrested. During the period covering 12 to 18 months post-baseline, three young adults were arrested (only one of whom was not also arrested in the previous period). In total, three young adults (14%) were arrested from baseline through 18 months after baseline. One young adult was arrested three times, a second young adult was arrested twice, and the third young adult was arrested once during this period. Thus, 86% of young adults who received MDFT had no rearrests during the 18-month study period. These arrest rates compare favorably to extant evaluation and research findings on criminal drug court participants, which shows reductions in rearrest rates ranging from 8 to 53%.^61,62^ Recall that 100% of the sample had been arrested prior to drug court.

## Discussion

The primary question of this study was whether it would be feasible and promising to extend a family-based intervention originally designed for adolescents to a different developmental period, namely that of young adulthood. Despite the growing evidence that family interventions are effective for both adolescents and adults, research and clinical practice have focused mainly on the role of the family in adolescent behavioral health and less on the role of the family with young adults or adults.^19,63,64^ Hogue et al. (2023) remind us that the lack of involvement of family in behavioral health is in sharp contrast to medical practice, where it is routine to involve family members.

In the present study, although concerned significant others such as siblings and romantic partners were not excluded from involvement in the therapy, all young adults invited their parents to participate in therapy. Both the young adult and their parent(s) participated in MDFT sessions and were helped to make important changes. MDFT focuses on how parents influence and interact with their children, not just on helping engage their young adult child into treatment and support their treatment process. Changing the parent’s attitudes and interactions with their young adult is a key path through which MDFT attempts to help the young adult reduce substance misuse and other problems. This contrasts with other interventions for young adults that focus on peers and the larger social network—not parents per se—as the primary source of influence.^17,64^ The authors speculate that widespread misconceptions are barriers to family, and especially parent, involvement in the treatment of young adults. For instance, unhelpful beliefs may include the following: that parents are more likely to exert a negative instead of positive influence on their young adult children, that the parent-child relationship loses its salience in the young adult years, and that the task for young adults is to develop autonomy and individuate away from their parents.^10,65,66^

Researchers approached this study with a decidedly different idea that parents have strong influence well beyond adolescence. With MDFT’s simultaneous work in multiple domains (young adult, parent, family, community), it was hypothesized to be particularly well suited to the young adult developmental period, and the results of this study concur. The findings support the premise that conducting family therapy with young adults and their parents together—promoting change with both—is feasible and shows promising effects. Specifically, 88% of the young adults agreed to enroll in MDFT with their parents. The intervention was delivered with strong fidelity, participant satisfaction was high, and over 95% of participants completed treatment. With respect to clinical outcomes, the results are very promising. Analyses revealed statistically significant decreases in substance use, arrests, and legal risk, as well as significant improvements in vocational functioning and employment status. Large effect sizes emerged for six of seven variables measured (over .70), and only one variable (ASI Composite Employment Scale) yielded a nonsignificant result. Overall, the results compare favorably with other intervention studies on young adults.^12,16,17^

### Strengths and limitations

Whereas the results are encouraging, this is a small feasibility study. Several limitations are evident and additional research is needed to fully evaluate the application of MDFT to young adult populations. The single group design and small sample size limit any definitive conclusions to young adults more broadly. The study took place in one criminal drug court, and results are not necessarily generalizable to other court or treatment settings. Regression to the mean is a possible contributor to young adults’ favorable outcomes over time, as they began the study at a crisis point in their lives. Finally, the sample was primarily male (71%) and Hispanic (62%), and hence, the results may not easily generalize to females and young adults from other ethnic groups.

The present study is a strong beginning, demonstrating the feasibility and promise of MDFT for young adults. Based on these results, randomized clinical trials of MDFT with more diverse samples of young adults examining multiple outcomes (substance use, mental health, educational/vocational functioning, legal risk) and longer follow-up periods are warranted.

## Implications for Behavioral Health

The findings from this study have several important implications for improving the delivery of behavioral health services to young adults. First, the results suggest that intensive multicomponent family therapy programs such as MDFT are feasible and beneficial to young adults and their parents. The results imply that not only should MDFT be implemented with this population, but also highlight the benefits of effective family therapy models to improve behavioral health among young adults. Despite evidence supporting family involvement in behavioral health care generally and substance use treatment in particular,^13,19,21,22,65^ very few individuals receive family therapy. For example, a DHHS-sponsored annual survey on the treatments used most frequently by providers did not even include family-based approaches in its list of 15 options.^20^ Family involvement is needed not just in the treatment of young adults, but in behavioral health care generally. The results point to the benefit of active engagement of parents in treatment—and motivating parents and other family members to change.

Given the success of family therapy for adolescent problems^19^ and the unprecedented need for behavioral health services among young adults,^67^ adaptation of evidence-based family therapy for this group seems a positive next step. Young adulthood is a distinctive developmental period of both risk and opportunity. For too many vulnerable young adults, risk overwhelms opportunity.^7,10,11^ Without effective treatment, many of these young adults are at extreme risk of a lifetime of challenges including overdose, accidents, mental health instability, unemployment, and incarceration. Young adults are undoubtedly in need of specialized treatment services given their high risk for offending, misuse of substances including opioids, and serious mental health problems.^7^ The need for innovative treatment for this age group is undeniable.^21^ Results from this study suggest that developmentally appropriate family-based interventions such as MDFT may be ideally suited for young adults.

Finally, given the strong feasibility and outcome results of this pilot study, one cannot help but speculate that perhaps MDFT or similar treatments might also benefit adults over the age of 25. Family influence generally, and parent influence in particular—for better or worse—does not necessarily wane in adulthood. The results of this study, coupled with the pioneering work of Stanton and Todd,^26^ suggest that family therapy is not only indicated for children and adolescents but also might be a beneficial adult treatment for substance use disorders and associated challenges. Thus, more research on applying MDFT and other family therapy models to adult populations is warranted. One innovative example is underway in Connecticut. The Connecticut Department of Children and Families recently received a grant from the Department of Health and Human Services to partner with Chestnut Health Systems in conducting a randomized clinical trial that combines Multidimensional Family Therapy and Multidimensional Family Recovery.^68^ MDFR is an evidence-based family approach found to be effective in engaging parents at risk for losing their children due to substance misuse. The integrated Multidimensional Family Therapy and Recovery program (MDFTR) treats parental substance misuse and co-occurring behavioral health challenges among child welfare involved parents. The results of this new study may inform the design and implementation of comprehensive family-based treatments for substance misuse and related problems well into adulthood.
